# Changing the culture is a marathon not a sprint

**DOI:** 10.1186/s13223-019-0325-6

**Published:** 2019-02-19

**Authors:** Jenna Dixon, Susan J. Elliott

**Affiliations:** 1 The School of Public Health and Health Systems, University of Waterloo, 200 University Ave W., Waterloo, ON Canada; 2 Geography and Environmental Management, University of Waterloo, 200 University Ave W., Waterloo, ON Canada

**Keywords:** Food allergy, Knowledge translation, Outcome measures, Patient engagement

## Abstract

Integrated knowledge translation (IKT) is built upon the premise that involving knowledge users as partners in the research process will result in science that is more relevant to the public and therefore will have greater impact. Drawing on our experiences with a large and multifaceted IKT food allergy research program we highlight the disjuncture between the goals of IKT and the nature of basic science research, most notably the long timelines before research is ready for translation. Our partner consultations concluded that IKT success should be measured in a different way. That is, it should not be about informing an immediate gap in the translation of food allergy findings but about building relationships between our partners, greater awareness, understanding and knowledge about the nature of science and IKT, and ultimately helping to create better policy and science down the road. It is the recognition that it behooves us as scientists to be able to answer those “why” questions. We call for other researchers to consider the success of IKT beyond the short term timelines of any one research project but instead as an avenue to build partnerships, innovate thinking about research questions and to maximize choice and minimize risk for individuals in Canada and beyond affected by food allergy.

To the Editor,

Inherent to all of our activities as scientists is the belief (and dare we say hope?) that the evidence we create will in some way have a meaningful impact on the people ultimately affected by our research. That is, we are not all tinkering away in our labs for our own good; as a community we move towards a greater knowledge base and this knowledge base can be of use to someone, somewhere. How and in what form we imagine this impact can of course vary widely from one research project to the next. Across the world, funding agencies, universities, scientists, clinicians and the public alike have been grappling with how we conceptualize, measure and value research that has impact. At the Canadian Institutes of Health Research this is coined as knowledge translation (KT) and is defined as “a dynamic and iterative process that includes synthesis, dissemination, exchange and ethically-sound application of knowledge to improve the health of Canadians” [1]. To Canadian credit, this definition has also been adopted by the World Health Organization [2, 3]. To the readers of this journal the term may evoke a range of ideas as well as feelings and, indeed, the definition of successful KT may look very different from one research project to the next.

For the past 5 years our work has been to execute the KT arm of a large Canadian food allergy research project known as GET-FACTS (Genetics, Environment and Therapies: Food Allergy Clinical Tolerance Studies). Instead of looking to traditional dissemination (end-of-grant) based KT approaches this project envisioned linking knowledge users at the knowledge creation stage [4] through a steering committee, and having them work *with* researchers as the science was developed. Essentially, this meant having knowledge users inform the basic science food allergy research agenda. This integrated KT (IKT) is built upon the premise that involving knowledge users as equal partners at all stages of the research process will result in science that is more relevant to knowledge users. There is a large and ever-growing literature that demonstrates the benefits of user engagement in research [5–7]. Throughout the course of our project we also measured the knowledge, attitudes and perceptions of both scientists and knowledge users towards the IKT model (the methods of which are described in detail elsewhere [8]).

A major finding of our work is one that is implicitly understood by almost all and yet rarely if ever explicitly stated in that growing body of IKT research: basic science does not always lend itself well to public involvement. To truly understand why this is the case one must ask, who are the knowledge users, who are the researchers and ultimately what are our measures of success? The IKT literature is dominated by examples of professional-level knowledge users (e.g. social services, health managers, politicians, public health) working alongside applied research disciplines (e.g. clinicians) [9–13]. IKT research has to date focused on either clinical, health service or population health research where the research goals lend themselves to knowledge user engagement because the application of that knowledge is clear and immediate.

Why is basic science different? We know that the translation goals with basic science are truly far down the road, usually much longer than the time-scale of any one project. While data are generally sparse and estimates vary based on how end-points are conceptualized, basic science research can tilt close to two decades in the making before it is found in practice [14]. For this reason, the general public is not usually considered an immediate stakeholder in this type of research [15]. Though biomedical research makes up the majority of health research applications received and funded in Canada [16] applied health sciences are far more involved with KT broadly [17] and IKT specifically.

Our project involved many different types of scientists engaged in many different types of science related to food allergy and, for the most part, those who were most comfortable with IKT were those who were already working closely with patients and other knowledge users in their work [8]. While the basic scientists may have been positive about the prospect of IKT, they understandably had difficulty imagining a patient sitting with them at the bench helping to conduct experiments. It is clear that the food allergy community cannot expect short term returns on their engagement with the basic science and, as a project, we realized that we had to think differently about how we measured the success of our IKT. Further, to define, measure and strive for these IKT goals we needed a comprehensive plan. So, our scientists and steering committee undertook a multi-year consultative process of developing a Performance Measurement Framework for our IKT [18]. We concluded that IKT success was not about informing an immediate gap in the translation of food allergy findings or changing intricate and long planned out scientific experiments but *instead* was about communication and education, networking and relationship building and evaluation and accountability. Or to say otherwise, it was about building relationships between our partners, greater awareness, understanding and knowledge about the nature of science and IKT, and ultimately helping to create better policy and science down the road. This is not in any way the elimination of pure science, but the recognition that it behooves us as scientists to be able to explain to users “why…”.

Our research tells us that those participating in this project have improved their understanding of what IKT is and we have met key deliverables which formed the basis of a carefully designed-in-relationship terms of reference for this project (e.g. *#6 “researchers have a better understanding of the role, and the importance of, knowledge translation in research”, #9 “researchers understand the process involved in the co*-*production of knowledge”, and #10 “researchers feel more empowered to contribute to the knowledge translation process”*). In our most recent round of data collection, a mid-point quantitative survey and qualitative semi-structured interviews, both scientists and steering committee members self-reported an increased knowledge of the role of IKT in research. While IKT may be time consuming and slow to start, we are now seeing some of those deliverables described above come to fruition. But the true success of our work will come long after GET-FACTS has run its course. Our work, and what we hope will be a call for other scientists, is a long term game looking to change the culture of science in order to think about research in a new light, to ask the burning questions that are relevant to knowledge users and, as stated in our terms of reference, to maximize choice and minimize risk for individuals in Canada and beyond affected by food allergy.

## Abbreviations

KT: knowledge translation
IKT: integrated knowledge translation
